# Influences of fluctuating nutrient loadings on nitrate-reducing microorganisms in rivers

**DOI:** 10.1093/ismeco/ycae168

**Published:** 2024-12-24

**Authors:** Shengjie Li, Rui Zhao, Shuo Wang, Yiwen Yang, Muhe Diao, Guodong Ji

**Affiliations:** Key Laboratory of Water and Sediment Sciences, Ministry of Education, Department of Environmental Engineering, Peking University, Beijing 100871, China; Max Planck Institute for Marine Microbiology, Bremen 28359, Germany; Department of Earth, Atmospheric and Planetary Sciences, Massachusetts Institute of Technology, Cambridge, MA 02139, United States; Key Laboratory of Water and Sediment Sciences, Ministry of Education, Department of Environmental Engineering, Peking University, Beijing 100871, China; College of Animal Science, South China Agricultural University, Guangzhou 510642, China; College of Environmental Science and Engineering, Tongji University, Shanghai 200092, China; Key Laboratory of Water and Sediment Sciences, Ministry of Education, Department of Environmental Engineering, Peking University, Beijing 100871, China

**Keywords:** nitrate reduction, chemoautotrophic denitrification, omics, microbial interaction, transcriptome

## Abstract

Rivers serve important functions for human society and are significantly impacted by anthropogenic nutrient inputs (e.g. organic and sulfur compounds). Reduced organic and sulfur compounds influence the nitrogen cycle as they are electron donors of microbial nitrate reduction. Water pollution caused by individual nutrients and the mechanisms have been studied, but how the variation in multiple nutrient loadings influences nitrate-reducing microorganisms is less understood. Two sets of microcosms were established and exposed to nitrate, along with either acetate or thiosulfate, at different times. Nutrient concentrations responded to the loading pollutant. The nutrient loading order was more important in shaping microbial community structure and microbial interactions through the exchange of growth-required substances. This indicated that upstream or historical nutrient inflows impacted current nitrate reduction by changing the seeding microbial community, highlighting the importance of river connectivity. Based on metatranscriptome analysis, although the order and type of nutrient loadings were equally important in regulating global transcriptomes, transcripts of enzymes for key metabolisms (nitrate reduction, sulfur oxidation, etc.) more actively responded to the nutrient type. The regulation of a small set of genes was sufficient to make the transition, while most transcripts were not degraded and regenerated. These insights are important for understanding the varying pollution status of rivers and for developing effective solutions, such as remediation.

## Graphical Abstract

**Graphical Abstract:**
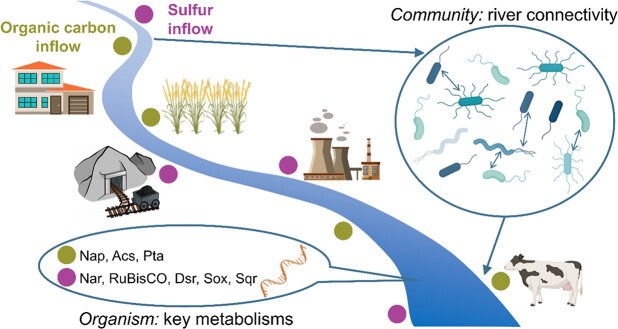

## Introduction

The discharge of bioavailable nitrogen (N) into continental waters has caused eutrophication, hypoxic zone development and harmful algal blooms around the globe [1, 2]. Despite enhanced removal by wastewater treatment, nutrient discharge to surface waters will likely continue to increase over the next decades [3, 4]. Rivers serve as dynamic conduits, connecting inland aquatic systems, linking terrestrial waters to the ocean, and transforming nutrients along their course. They are important ecosystems for humans, providing clean water and serving diverse functions such as transportation, hydroelectricity, flood control, and recreation. Thus, N pollution in rivers poses a great threat to the human society [5, 6].

Nitrate is one of the major forms of N in inland waters [7]. Its removal from aquatic ecosystems is mainly mediated by microorganisms via nitrate reduction [8]. Nitrate-reducing bacteria can be heterotrophic (i.e. use organic compounds as the electron donor) and chemoautotrophic (i.e. depend on reduced inorganic substances as the electron donor, such as sulfur) [9, 10]. *Pseudomonas*, *Escherichia*, *Klebsiella*, and *Citrobacter* are commonly observed heterotrophic nitrate reducers [11, 12], whereas *Thiobacillus* and *Sulfurimonas* are typical sulfur-dependent nitrate reducers [13, 14]. Nevertheless, a couple of organisms, such as *Pseudomonas* and *Thauera*, use both types of electron donors and thrive in either organic- or sulfur-rich environments [15]. The end product of nitrate reduction could be nitrogen gases, produced by denitrification, or ammonium, produced by dissimilatory nitrate reduction to ammonium (DNRA). The competition between the two pathways is influenced by the type of nutrients, i.e. organic and sulfur compounds [16, 17]. Rivers are exposed to multiple point sources of nutrient pollutants, including organic matters in domestic sewage and agricultural runoff, as well as sulfur compounds from mining wastewater and industrial discharge [18–23]. These pollutants could foster the presence and metabolism of both types of nitrate-reducing microorganisms. Previous experiments used stable nutrient loadings throughout the incubations [24–26], while the fluctuations and interactions between different pollution types and sites are rarely studied. We do not yet understand how nitrate reduction pathways and associated microorganisms coordinate with fluctuations in multiple nutrient loadings.

Water connectivity, which refers to the movement and exchange of materials within a water body between different locations, is a basic attribute of rivers and is also a fundamental parameter for assessing ecosystem health [27]. Microorganisms in rivers may be influenced by both current and upstream nutrient loadings. At the community level, attempts have been made to understand how nutrient types shape microbial community assembly in laboratory cultures, which used a single type of nutrients throughout the incubations [11, 28]. Other studies observed legacy effects on microbial community, where the particular order and timing in which different species happen to colonize decide the community structure [29, 30]. However, the influences of current nutrient loadings and historical events on microbial community have not been compared. At the single population level, model organisms have been used to study microbial responses to environmental change. The studied microorganisms may (i) up- or down-regulate gene transcripts according to the changes [31–33], (ii) exhibit heterogenous phenotypes to defend against possible future changes [34, 35], or (iii) express a combination of genes that enable them to deal with different conditions [36, 37]. How current nutrient loading types and upstream or historical nutrient loadings influence gene expression of nitrate-reducing microorganisms remains unknown. A mechanistic understanding of community assembly and microbial gene expression in response to nutrient loadings is needed to rationally manipulate responsible microorganisms towards beneficial states.

With the background of complex pollution sources, we aimed to investigate the influences of fluctuations in multiple nutrient pollutants on nitrate-reducing microorganisms in riverine systems. Microcosm incubations were inoculated with river samples and fed with nitrate. According to previous studies, acetate is a major product of organic matter degradation [38], and thiosulfate is a common form of sulfur in mining wastewater [39]. Fluctuating input of acetate and thiosulfate with inverted orders, were used in two experiments to simulate the inflows of organic carbon and sulfur in rivers, respectively. Microbial community assembly processes were monitored using 16S rRNA gene amplicon sequencing. Established communities and enriched populations were analyzed with genome-centered metagenomics and metatranscriptomics. We hypothesized that (1) different nutrient loading histories with the same types of nutrients would initially result in distinct microbial communities but ultimately converge to a similar community structure, and (2) nitrate-reducing microorganisms would respond to the type of nutrient loading at either the community or transcript level.

## Materials and methods

### Experimental setup

Water and sediment samples were collected from Guangfu River, Jining, Shandong, China. The water and sediment samples were mixed in a 1:1 ratio and manually stirred. 20 g of the mixed water-sediment sample was transferred into 500-mL serum bottles containing an inorganic basic medium. The total volume of the sample and the medium in serum bottles was 400 mL. The basic medium contained MgCl_2_ • 6H_2_O (2 mM), CaCl_2_ (0.9 mM), KH_2_PO_4_ (11 mM), NH_4_Cl (0.5 mM), NaHCO_3_ (10 mM), trace element solution (1 mL), and vitamin solution (1 mL) according to a previous study [40].

Two microcosm experiments were set up and exposed to different nutrients at different times (Fig. S1). Both experiments went through a total of eight phases, including four phases with acetate addition (C) and four phases with thiosulfate addition (S) in addition of nitrate addition. In the first experiment (named “CS”), 5-mM nitrate (final concentration, the same for other nutrients) and 5-mM acetate were added at the beginning of the first phase, while 5-mM nitrate and 5-mM thiosulfate were added at the beginning of the second phase. This pattern continued for the subsequent phases, resulting in the order of nutrient inputs being C-S-C-S-C-S-C-S. In the other experiment (named “SC”), the order of nutrient inputs was inverted to S-C-S-C-S-C-S-C. Briefly, 5-mM nitrate and 5-mM thiosulfate were added at the beginning of the first phase, while 5-mM nitrate and 5-mM acetate were added at the beginning of the second phase. This pattern continued for the next six phases.

Triplicated systems were set up for each experiment. Each phase ended when nitrate concentration in all systems was below detection. At the start of each phase, 200 mL of culture was replaced with 200 mL of fresh basic medium, and the systems were flushed with nitrogen to maintain anoxic conditions. Rubber stoppers and aluminum crimp caps were used to seal the systems. The systems were run in the dark for a total of 37 days.

### Sampling and nutrient measurements

**Figure 1:**
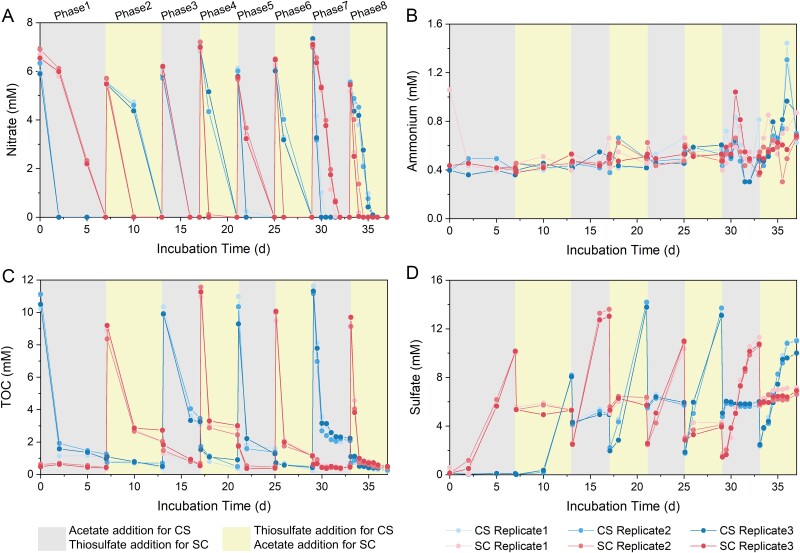
Nutrient dynamics in the experiments. Concentrations of (A) nitrate, (B) ammonium, (C) TOC and (D) sulfate in the CS and SC experiments. Both experiments comprised eight phases, with phases using acetate (C) and thiosulfate (S) indicated with different colors.

Culture samples were collected from the experiments at the start, midpoint and end of each phase (Fig. 1). The sampling and nutrient concentration measurements were performed according to our previous methods [15]. In brief, 5-mL cultures were collected from the systems using syringes and centrifuged. The pellets were used for molecular analysis, such as DNA/RNA extraction and sequencing. The supernatants were filtered through 0.2-μm membranes for nutrient measurements. Nitrate and sulfate concentrations were measured with an ion chromatograph ICS-1100 (ThermoFisher, California, USA) equipped with an anion-exchange column (Dionex IonPac AS23; 4 × 50 mm^2^; Thermo Scientific). Nitrate reduction rate was calculated (Supplementary Method). Ammonia was measured using the indophenol reaction [41] with a spectrophotometer (Shimadzu, Japan) at the wavelength of 625 nm. Total organic carbon (TOC) content was measured using a TOC analyzer (TOC-L CPH, Shimadzu, Japan).

### DNA extraction, amplicon sequencing and analysis

Samples collected at the start of the experiment and at the end of each phase were used for amplicon sequencing analysis. Amplicon sequencing and analysis were performed according to a previously described workflow [42]. First, DNA was extracted using the FastDNA Spin Kit for Soil (MP Biomedicals, USA). The 16S rRNA gene was amplified with the primers 338F and 806R [43] using polymerase chain reaction. The products were paired-end sequenced on the Illumina MiSeq sequencing platform using PE300 chemicals. Raw reads were merged with FLASH v1.2.11 [44] and quality-filtered with fastp v0.19.6 [45]. Quality-controlled sequences were further analyzed with QIIME 2 v2020.2 [46].

### Metagenomic sequencing and analysis

Samples collected from each system at the end of the last acetate phase and the last thiosulfate phase (Phase 7 and 8 in Fig. 1) were used for shotgun metagenomic sequencing, resulting in a total of 12 samples (2 experiments, 2 phases, and 3 replicates). Sequencing was performed as previously described [42]. In brief, DNA samples were fragmented to ~400 bp and used for paired-end library construction. Adaptors were ligated to the ends of DNA fragments, and the libraries were sequenced on the Illumina NovaSeq 6000 platform using NovaSeq Reagent Kits.

Raw reads were quality-filtered with BBDuk (Bushnell B., github.com/BioInfoTools/BBMap/blob/master/sh/bbduk.sh). Reads were co-assembled and individually assembled with MEGAHIT v1.2.9 [47]. Filtered reads were mapped to the assembled contigs with a 99% identity cut-off using BBmap v38.18 (Bushnell B., sourceforge.net/projects/bbmap/). The coverage of assembled contigs was calculated with the “jgi_summarize_bam_contig_depths” script within MetaBat v2:2.15 [48]. Metagenome-assembled-genomes (MAGs) were obtained from different libraries with MetaBat v2:2.15 [48] and dereplicated with dRep v3.2.2 [49]. Characteristics of the MAGs were estimated with CheckM v1.1.3 [50]. Taxonomic information of the MAGs was obtained with GTDB-Tk v1.0.2 [51]. Redundant contigs from different libraries were removed with cd-hit v4.8.1 [52]. The average nucleotide identity between MAGs was calculated with FastANI v1.33 [53]. The relative sequence abundance of each MAG was calculated by dividing the sequencing coverage of the MAG by the sum of sequencing coverages of all non-redundant contigs. MAGs were annotated using Prokka v1.13 [54], METABOLIC v4.0 [55] and MetaErg v2.3.39 [56]. The presence of denitrification genes was checked using Hidden Markov Models v3.3.2 [57] following a previously described protocol [14]. Genes were also annotated against the KEGG database (genome.jp/kegg/). Biosynthesis of amino acids and vitamins was confirmed based on KEGG pathway maps. Transporters were identified with TransAAP (membranetransport.org/transaap) [58]. Sequencing coverage of each contig was normalized against the average sequencing coverage of all contigs.

### RNA extraction, metatranscriptomic sequencing and analysis

Metatranscriptomes corresponding to the 12 metagenome samples were collected. RNA extraction was preformed using the E.Z.N.A.® Soil RNA Midi Kit (Omega Bio-tek, Norcross, GA, USA). The quality of extracted RNA products was assessed using 1% agarose gel electrophoresis and an RNA6000 Nano chip on an Agilent 2100 Bioanalyzer. rRNA was removed using the Ribo-zero Magnetic kit (Epicentre, Illumina). Libraries were prepared using the TruSeq™ RNA Sample Prep Kit (Illumina) and sequenced on the Illumina HiSeq 2500 platform using HiSeq 4000 PE Cluster Kit and HiSeq 4000 SBS Kits.

Sequencing reads underwent trimming of the 3′ and 5′ ends using SeqPrep (John J., github.com/jstjohn/SeqPrep) and removal of low-quality reads using Sickle (Jochi N. A. and Fass J. N., github.com/najoshi/sickle). rRNA sequences were further removed with SortMeRNA v2.1b [59]. Quality-filtered reads were mapped to the non-redundant contigs using BBmap v38.18 (Bushnell B., sourceforge.net/projects/bbmap/) with a 99% identity cut-off. The relative sequence abundance of MAGs in metatranscriptomes was calculated using the same method as in metagenomes. Sequencing depth of a gene was determined with HTSeq v1.99.2 [60]. Transcripts per million (TPM) of a gene in a MAG were calculated based on the transcriptome of the MAG to remove the influence of the relative abundance of the MAG in metatranscriptomes. The global transcriptome difference of a MAG between phases, replicates or experiments was calculated as the percentage of transcripts that differed, as previously described [61]. Only transcriptomes with more than 100 reads mapped in all samples were included in this calculation.

### Statistical analysis

Non-metric multidimensional scaling (NMDS) was performed with the “dplyr” and “vegan” packages in R v4.0.5, based on Bray–Curtis distances. Samples were grouped in NMDS plots using the “ordiellipse” function from the “vegan” package. For the comparison of nutrient concentrations and population abundances between phases, experiments, and replicates, *t*-tests were conducted in PASW Statistics v18.0. Statistical significance was defined as *P*-values < 0.05.

## Results

### Nutrient loadings and microbial performance

Two microcosm experiments were inoculated with river samples containing abundant nutrients (0.088-mM nitrate, 0.59-mM TOC, and 5.5-mM sulfate). Each experiment proceeded through eight phases, alternating between four phases with acetate addition (C) and four phases with thiosulfate addition (S), with the order of C/S additions reversed between the two experiments (Fig. S1). During the initial two phases, nitrate was fully consumed within three days during the C phases of both experiments, while during the S phases nitrate could not be reduced within such a short time (Fig. 1A, Supplementary Table 1). Subsequently, nitrate was consumed within 4 days during both C and S phases. At the start of the final two phases, a faster nitrate reduction rate was always observed during the C phases than the S phases, regardless of the experiments (Fig. S2, Supplementary Table 2). Ammonium did not exhibit a clear pattern during the first six phases (Fig. 1B). However, during the final two phases, ammonium concentrations were higher at the S phases compared to the corresponding C phases in both experiments. The active use of acetate was revealed by the decrease in TOC of about 8 mM during each C phase (Fig. 1C). At the end of each C phase and the start of each S phase, TOC decreased by half due to culture replacement with fresh medium in half the volume. Approximately 10-mM sulfate was produced from thiosulfate oxidation during each S phase (Fig. 1D), quantitatively consistent with the stoichiometry of thiosulfate oxidation to sulfate. From the third to the eighth phases, we still observed a decrease in TOC even during the S phases, and an increase in sulfate during the C phases, indicating that the enriched microbial communities used both acetate and thiosulfate as electron donors. The change in nutrient concentrations was in pace with the C/S phase alternations, indicating successful establishment of nutrient loading dynamics in the experiments.

### Microbial community assembly

**Figure 2:**
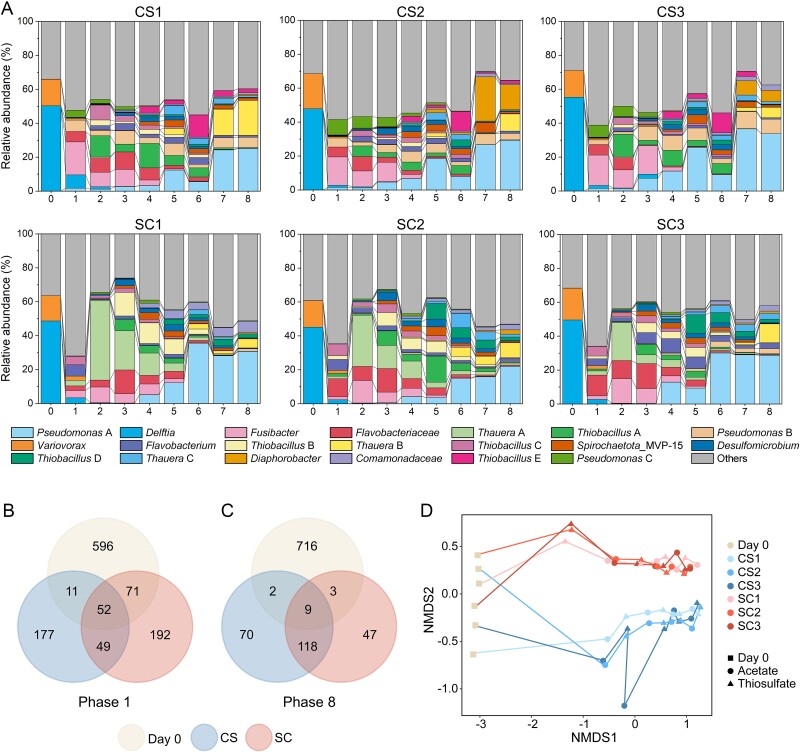
The order of nutrient loadings is more important in microbial community assembly. (A) Change in relative abundances of the 20 most abundant populations (amplicon sequence variants, ASVs) over eight phases. The triplicates are shown individually in each row. (B, C) Number of ASVs shared between the inoculated community and enriched communities at the end of the first phase (b) and the eighth phase (C). (D) NMDS of all samples collected in the experiments based on ASV analysis. Samples collected from the same systems are connected in a time series.

Based on 16S rRNA gene amplicon sequencing data (Supplementary Table 3), *Delftia* and *Variovorax* amplicon sequence variants (ASVs) were the two main residents in the original river samples (Fig. 2A). With acetate or thiosulfate additions, populations affiliated with *Fusibacter* and *Flavobacteriaceae* were enriched during the first to the fourth phases in all incubations. During the last four phases, “*Pseudomonas* A” was the most abundant population. Other enriched species at the end of the incubations were affiliated with *Thauera*, *Diaphorobacter*, and *Comamonadaceae*. *Pseudomonas* and *Thauera*, previously enriched in the presence of both acetate and thiosulfate, are known as mixotrophic nitrate reducers [15]. Among the twenty most abundant populations, seven populations showed significantly different relative abundances between the two experiments, whereas no population showed significantly different relative abundances between the two phases (Supplementary Table 4). For example, “*Thiobacillus* D” was mainly present in SC, while “*Thiobacillus* E” was present in CS.

The enriched communities diverged obviously from the initial river community. Microbial communities had already largely changed by the first nutrient inflow, which weeded out 596 ASVs from the systems (Fig. 2B). Meanwhile, 101 ASVs were shared between the CS and SC experiments. As a result, the communities evolved along two directions, shown in the NMDS analysis (Fig. 2D). An additional 120 ASVs were lost during subsequent nutrient loadings (Fig. 2C). During the following nutrient loadings, the percentage of species shared by the original inoculum and the CS/SC experiments decreased, while the percentage of species shared by the two experiments increased. Consistently, the two experiments became more similar from the second loading phase onwards (Fig. 2D). Nevertheless, at the end of each nutrient loading phase, samples collected from the three replicates of an experiment clustered closely together, while samples from the two experiments were distinct. Overall, the order of nutrient loadings played a more important role in shaping community assembly than the specific acetate/thiosulfate phases.

### Enriched microorganisms

By shotgun metagenome sequencing, assembly and genome binning, we obtained a total of 69 MAGs (Supplementary Table 5). They collectively accounted for over 99.9% of the community in all metagenomes, and over 98.4% of the community in all metatranscriptomes. Among them, 51 MAGs were affiliated with *Gammaproteobacteria*, including 49 MAGs affiliated with *Burkholderiales* (12 *Giesbergeria*, 10 *Thiobacillus*, 9 *Azonexus*, 6 *Diaphorobacter*, 3 *Desulfobacillus*, and 3 *Thauera* populations, Fig. S3). Other *Gammaproteobacteria* organisms included *Alicycliphilus*, *Rhodocyclaceae*_UBA2250, and *Thiobacillaceae*_UBA6918. An alphaproteobacterial bacterium, *Tabrizicola*, was also observed. Other MAGs were affiliated with the phyla *Bacteroidota* (*n* = 9), *Bacillota* (*n* = 4), *Riflebacteria* (*n* = 2), *Desulfobacterota* (*n* = 1), and *Patescibacteria* (*n* = 1).

**Figure 3:**
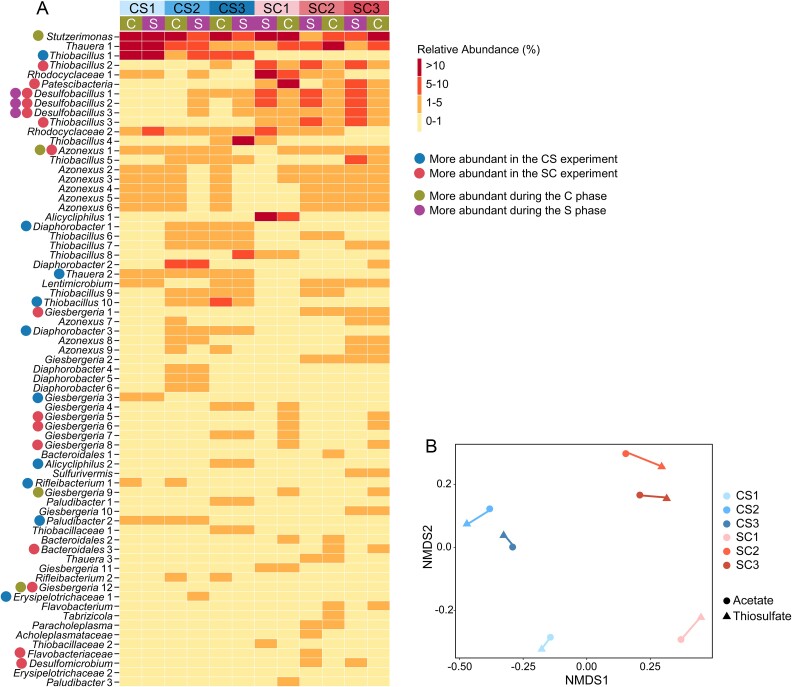
The order of nutrient loadings is more important in shaping the enriched community structure. (A) Relative abundance of populations each associated with a MAG during the final two phases. The top bar indicates the experiment (CS or SC), and the second bar indicates the acetate (C) or thiosulfate (S) phase. Populations with significantly higher relative abundance at acetate/thiosulfate phases or in CS/SC experiments are indicated with dots in difference colors. (B) NMDS based on the relative abundance of MAGs. Samples (dots and triangles) collected from the same systems are connected using lines.

The most abundant populations were “*Stutzerimonas*” (“*Pseudomonas* A” in 16S rRNA gene amplicon sequences) and “*Thauera* 1” (“*Thauera* B” in 16S rRNA gene amplicon sequences), which showed high relative abundances in all 12 metagenomes (Fig. 3A). For other less abundant populations, sometimes members affiliated with the same genus showed consistent trends across the two experiments and phases, while sometimes they did not. For example, all three members of *Desulfobacillus* (“*Desulfobacillus* 1”, “*Desulfobacillus* 2”, and “*Desulfobacillus* 3”) were more abundant in SC than in CS (*P* < 0.05, Supplementary Table 6). They were also significantly more abundant during the thiosulfate phase compared to the acetate phase (*P* < 0.05). However, some members within the same genus showed distinct patterns between the two experiments. For example, “*Thiobacillus* 1” had an average abundance of 9.2% in CS but only 0.051% in SC. “*Thiobacillus* 2” had an average abundance of 0.70% in CS versus 5.5% in SC, while the average relative abundance of “*Thiobacillus* 3” was 0.60% in CS and 4.1% in SC. “*Thiobacillus* 2” and “*Thiobacillus* 3” shared 98.8% genome identity, whereas they shared 81.6%–82.4% identity with “*Thiobacillus* 1” (Supplementary Table 7). In summary, the relative abundances of 25 MAGs differed significantly between the two experiments, while 7 populations showed significant differences between the two phases (Supplementary Table 6). NMDS analysis based on MAG relative abundances showed that samples from the same experiments clustered more closely than samples from the same acetate/thiosulfate phases (Fig. 3B). Overall, the influence of nutrient inflow order was more important in shaping the community compared to specific acetate or thiosulfate inflows, consistent with amplicon sequencing results.

### Global transcriptome regulation

To assess the activity of each MAG in the microcosms, we assessed their gene expression using the transcriptomes, where TPM values were calculated based on all genes present in a MAG. To compare the effects of the acetate/thiosulfate nutrient type and the nutrient loading order, we calculated the percentage of transcripts that changed between phases and experiments. Additionally, to assess the influence of microbial stochastic behaviors and experimental bias, transcriptome differences between replicates were calculated. Changes between the acetate phase and the thiosulfate phase within an experiment reflected the effect of the nutrient loading type, whereas differences between the SC experiment at the acetate phase and the CS experiment at the acetate phase reflected the effects of the nutrient loading order. In parallel, differences between any two replicates at the same phases provided insight into stochastic transcriptome changes.

**Figure 4:**
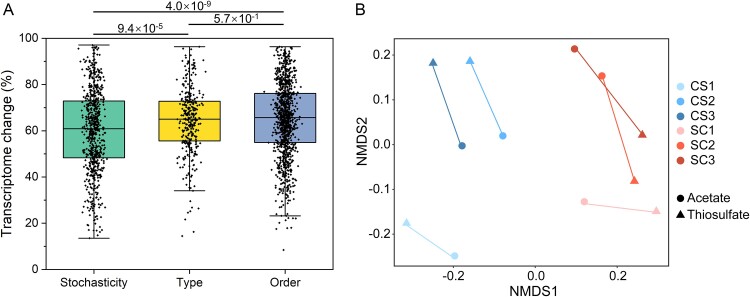
The order and type of nutrient loadings are similarly important in modulating global transcriptomes. (A) Influence of stochasticity, nutrient type, and order on global transcriptomes. Each dot represents a transcriptome turnover between replicates (Stochasticity), phases (Type), or experiments (Order) for a single MAG. Only transcriptomes with more than 100 reads mapped in all samples are included. Horizontal lines above the boxplot indicate pair-wise *P-*values. (B) NMDS based on global transcriptomes. Samples (dots and triangles) collected from the same systems are connected using lines.

The results indicated a significantly greater importance of the nutrient type and the nutrient order than microbial stochastic behaviors (Fig. 4). However, the influences of the type and order did not show significant differences. Though the nutrient input order was more important in community assembly and structure, the effects of the acetate/thiosulfate type and the nutrient input order were similarly important in regulating gene expression. Importantly, both factors exerted greater influence than stochastic transcriptome regulation (Fig. 4A).

### Microbial interactions

Microbial interactions through the exchange of growth-required substances, including amino acids [62] and vitamins [63], are important factors in determining community structure. We examined genes involved in the biosynthesis and transport of these substances in the genomes of the ten most abundant populations, excluding the *Patescibacteria* genome due to its reduced metabolism.

**Figure 5:**
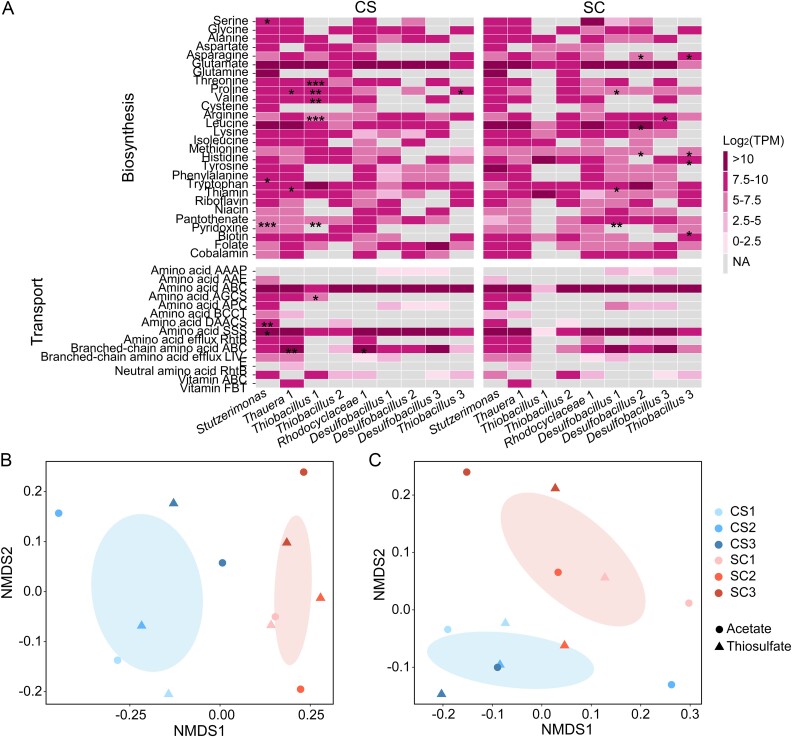
The order of nutrient loading is more important in shaping microbial interactions. (A) Average TPM values of genes involved in the biosynthesis and transport of amino acids and vitamins in the 10 most abundant populations (excluding *Patescibacteria*) across triplicated incubations. Genes with significantly higher TPM values in CS are indicated on the left side, while those with significantly higher TPM values in SC are indicated on the right side. *P*-values <0.05, < 0.01 and < 0.001 are indicated with “*”, “**”, and “***”, respectively. (B, C) NMDS based on genes involved in (B) the biosynthesis and (C) transport of amino acids and vitamins. Samples (dots and triangles) collected from the same experiments are grouped with ellipses.

“*Thauera* 1” and “*Rhodocyclaceae* 1” exhibited similar expression of these genes across phases and experiments (Fig. S4). In contrast, *Stutzerimonas* and “*Thiobacillus* 1” had fewer genes expressed in SC than in CS. In *Stutzerimonas*, downregulated genes mainly included those responsible for the biosynthesis of vitamins, such as niacin, pantothenate, pyridoxine, folate, and cobalamin. Despite the similar relative abundances of *Stutzerimonas* in CS and SC, it likely played a more important role in providing vitamins to other members and mediating community interactions in CS. In “*Thiobacillus* 1”, most genes related to the biosynthesis and transport of growth-required substances were less expressed in SC (Fig. 5A). In contrast, “*Thiobacillus* 2”, “*Desulfobacillus* 1”, “*Desulfobacillus* 2”, “*Desulfobacillus* 3”, and “*Thiobacillus* 3” showed increased gene expressions in SC (Fig. 5A). For example, “*Thiobacillus* 2” downregulated genes involved in the biosynthesis of leucine, pantothenate, and folate, as well as ABC transporters for branched-chain amino acids and vitamins. Average gene expression values associated with each abundant population in CS and SC were calculated (Fig. 5A). Among *Stutzerimonas*, “*Thauera* 1”, “*Thiobacillus* 1”, “*Thiobacillus* 2”, and “*Rhodocyclaceae* 1”, several genes were significantly more abundant in CS compared to SC, particularly those encoding enzymes for the biosynthesis of threonine, proline, valine, arginine and pantothenate in “*Thiobacillus* 1”. In “*Desulfobacillus* 1”, “*Desulfobacillus* 2”, “*Desulfobacillus* 3”, and “*Thiobacillus* 3”, some of these genes were significantly more abundant in SC. NMDS showed that samples from the same experiments were more similar based on gene transcripts involved in both the biosynthesis (Fig. 5B) and transport of amino acids and vitamins (Fig. 5C). These findings indicated a greater influence of the nutrient loading order on microbial interactions than specific acetate/thiosulfate phases.

### Regulation of key metabolic pathways

**Figure 6:**
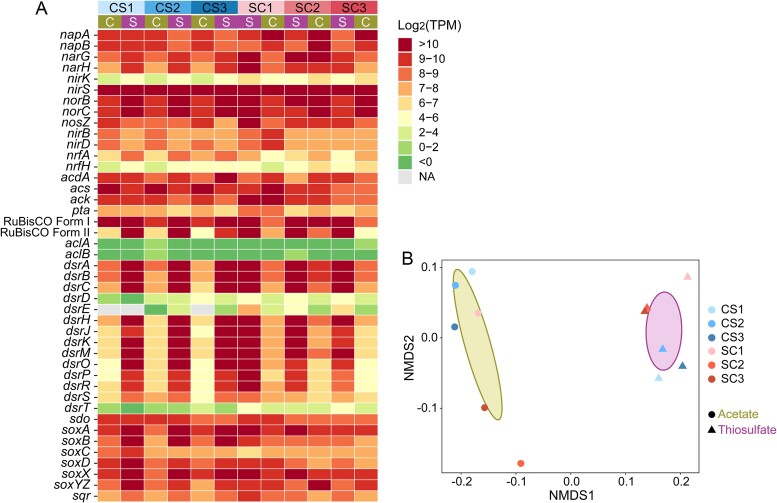
Microorganisms respond to nutrient loadings by regulating a few enzymes involved in key metabolic pathways. (A) TPM of functional genes involved in nitrate reduction, acetate transformation, carbon fixation, and sulfur oxidation. (B) NMDS based on transcripts of functional genes. Samples collected during phases with the same nutrient inflows (acetate or thiosulfate) are grouped.

To understand how microbial responses to nutrient phases were regulated, we focused on the transcriptional regulation of genes involved in a few key metabolic pathways, including nitrate reduction (via denitrification and DNRA), carbon fixation, acetate transformation and sulfur oxidation (Fig. 6). Though we observed equal importance of the nutrient loading phase and the nutrient loading order in regulating global transcriptomes, key functional genes displayed a more obvious pattern between phases than between experiments (Fig. 6A). Genes involved in sulfur oxidation (both the sulfide-quinone oxidoreductase system and the Sox system) and the Calvin cycle (two main forms of ribulose-1,5-bisphosphate carboxylase-oxygenases, RuBisCO) were upregulated during the thiosulfate phases compared to the corresponding acetate phases (Fig. 6A). In contrast, genes for acetate transformation, especially *acs* encoding the acetyl-CoA synthetase and *pta* encoding the phosphate acetyltransferase, were upregulated during the acetate phases (Fig. 6A). Even for genes involved in nitrate reduction, different expression patterns were observed with different nutrient types. For example, the Nap system (*napA* and *napB*) encoding the periplasmic nitrate reductase, was more highly expressed during the acetate phases, while the Nar system (*narG* and *narH*) encoding the cytoplasmic nitrate reductase was more actively expressed during the thiosulfate phases (Fig. 6A). This shows agreement with previous results that Nap was more important in heterotrophic nitrate reduction, while Nar was more prominent in chemoautotrophic nitrate reduction in freshwater lakes [15, 64]. The most abundant nitrite reductase encoding gene, *nirS*, was always highly expressed regardless of acetate or thiosulfate additions (Fig. 6A). The nitric oxide reductase Nor was more abundant during thiosulfate phases (Fig. 6A), which might indicate that N_2_O production in rivers could be increased in sulfur inflow. The nitrous oxide reductase NosZ showed different patterns in the two experiments, more abundant during the acetate phase in CS but during the thiosulfate phase in SC. Overall, clear distinctions between samples collected during acetate and thiosulfate phases were observed in NMDS (Fig. 6B).

## Discussion

The continuous and dynamic loading of nutrient pollutants, including organic matter, nitrogen and sulfur compounds, into rivers has become a global concern. In this study, we designed multi-nutrient variation experiments to explore the molecular mechanisms behind the dynamics in nutrients. The nutrient loading phase, involving the addition of acetate or thiosulfate, simulated different pollutants in rivers, allowing us to observe how microorganisms respond to these pollutants. The two experiments with different nutrient loading orders simulated distinct historical/upstream nutrient loadings. The difference observed between the two experiments reflected the influence of historical events on nitrate-reducing microorganisms. As the influence of historical events is linked to river connectivity, i.e. the movement of upstream nutrients and microorganisms to the current location, we interpreted the differences between the two experiments as the effect of river connectivity.

First we observed changes in nitrate reduction pathways. Organic matter was more efficient in nitrogen removal, while more ammonium was produced when thiosulfate was added (Fig. 1). The production of ammonium from nitrate reduction presents challenges to water remediation as bioavailable nitrogen accumulates in the water phase. The effects of sulfur compounds on the competition between denitrification and DNRA vary across environments [16, 26, 65], and the outcome may be linked to specific sulfur forms [66]. Further efforts are still needed to understand the mechanisms of how sulfur compounds stimulate denitrification or DNRA in different systems.

At the community level, the obtained MAGs represented over 99.9% of the enriched community, indicating our results could be applied to nearly the entire community. Most populations showed generalistic lifestyles, as they thrived during both acetate and thiosulfate loading phases. In systems experiencing frequent redox fluctuations, such as rivers and tidal areas, generalists tend to outcompete specialists in abundance [37, 61]. Interestingly, different generalists, e.g. the three *Thiobacillus* populations, thrived with different nutrient loading orders. Nucleotide divergence might contribute to this niche differentiation: two of the populations (“*Thiobacillus* 2” and “*Thiobacillus* 3”) shared a higher genome identity, and both of them were enriched in SC. “*Thiobacillus* 1” with a lower genome identity to others was more successful in CS. Future studies may investigate how these closely related populations are selected by different nutrient loadings.

Our results showed a greater impact of the nutrient loading order over the nutrient type on microbial community assembly and structure (Figs 2 and 3). Contrary to our first hypothesis, this effect was evident not only during the early stages but also in the structure of the final enriched microbial community. Therefore, microbial communities in rivers are shaped not only by current nutrient inflows but also by historical nutrient dynamics. The first nutrient loading is particularly important, as it selects certain organisms while eliminating others. Subsequent nutrient inflows drive stepwise adaptation in community structure. This is previously described as “legacy effects”: the historical order of nutrient loadings leads to different compositions of the final community, because early enriched populations gain advantage over others [67]. For example, the three *Desulfobacillus* populations were more abundant during the thiosulfate loading phase, and also more abundant in the SC experiment. They have already been selected during the first thiosulfate inflow, and this seeding community influenced the subsequent assembly process. A requirement for historical events to exert effective influence on microbial community is that the regional pool contains species that can together cause priority effects [68]. This regional pool might be shaped by microbial interactions [69, 70], so we investigated these interactions in regards of the exchange of growth-required substances, including amino acids and vitamins. Similarly, the nutrient loading order was more important in regulating microbial interactions than the nutrient type (Fig. 5). Future studies could evaluate how and to what extent historical factors influence microbial interactions.

These findings highlighted the importance of hydrological connectivity: upstream influxes, such as industrial wastewater, domestic sewage, tributary inputs, and rainwater runoff, influence microbial communities and the self-purification capacity in downstream water bodies. River management strategies should prioritize overall integrity and ecological connectivity. Developing an integrated approach is important for assessing ecological health, understanding variations in trophic states, and predicting future environmental conditions in hydrologically connected aquatic habitats. In designing future environmental studies, it is essential to consider the starting microbial community as it greatly influences the eventual microbial community structure and metabolic capacity.

Though the nutrient loading order was more important than the nutrient type in both community assembly and microbial interactions, microorganisms responded to nutrient phase rather than loading order, as reflected by changes in nutrient concentrations (Fig. 1). While global transcriptomes responded similarly to the phase and the order (Fig. 4), transcripts of key metabolic genes (i.e. nitrate reduction, carbon fixation, sulfur oxidation, and acetate transformation) responded more actively to the nutrient loading phase alternation (Fig. 6). This supported our second hypothesis: nitrate-reducing microorganisms responded to the nutrient loading at the transcript level. Interestingly, freshwater river microorganisms react to nutrient fluctuations mainly through the regulation of a small set of genes that are involved in key metabolic processes. This select set of genes is adequate for organisms to adapt to nutrient fluctuations, while overall transcriptomes remain relatively stable. Transcripts of other genes do not need to be degraded or reproduced during the change, which saves a lot of energy and helps microorganisms survive the fluctuation. These findings are important for designing molecular studies in dynamic habitats, where monitoring the expression and regulation of key enzymes should be prioritized.

## Supplementary Material

SI_ycae168

SI_ycae168

## Data Availability

All sequencing data generated in this study are available at NCBI (PRJNA848245). The BioSamples for the 16S rRNA sequences are SAMN28991586–SAMN28991639. The BioSamples for the MAGs are SAMN28993175–SAMN28993243. The BioSamples for the metagenome raw reads are SAMN28991640–SAMN28991651. The BioSamples for the metatranscriptome raw reads are SAMN28991652–SAMN28991663.
