# Supplementary material for: Influences of fluctuating nutrient loadings on nitrate-reducing microorganisms in rivers
Source: ISME Commun. 2024 Dec 24;5(1):ycae168. doi: 10.1093/ismeco/ycae168 (PMC11748280; doi:10.1093/ismeco/ycae168)
Supplement: SI_ycae168 [file si_ycae168.pdf]

Supporting Information for

Influences of fluctuating nutrient loadings on nitrate-reducing microorganisms in rivers

Shengjie Li [a, b], Rui Zhao [c], Shuo Wang [a], Yiwen Yang [d], Muhe Diao [e], Guodong Ji [a, *]

[a] Key Laboratory of Water and Sediment Sciences, Ministry of Education, Department of Environmental Engineering, Peking University, Beijing 100871, China
[b] Max Planck Institute for Marine Microbiology, Bremen 28359, Germany
[c] Department of Earth, Atmospheric and Planetary Sciences, Massachusetts Institute of Technology, Cambridge, Massachusetts 02139, USA
[d] College of Animal Science, South China Agricultural University, Guangzhou 510642, China
[e] College of Environmental Science and Engineering, Tongji University, Shanghai 200092, China

* Corresponding author.
Email address: jiguodong@pku.edu.cn

Supplementary Method

Nitrate reduction rate during the last two phases was determined as the slope of the linear fitting applied to nitrate concentrations from three consecutive samples at a given time point (Fig. S2). These three samples included the one collected just before the time point, the one collected at the time point, and the one collected just after the time point. At the start of each phase, the nitrate reduction rate was assumed to be 0. Additionally, when nitrate concentration fell below the detection limit, the nitrate reduction rate was also considered to be 0.

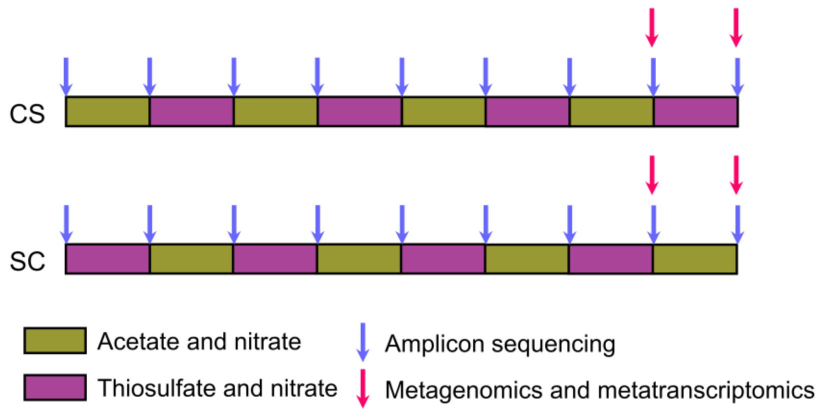

Fig. S1 Nutrient loading design of this study. Alternating phases of acetate and thiosulfate additions were built in the two nitrate-reducing microcosm experiments with inverted addition orders. Nutrient measurements and amplicon sequencing were performed at the beginning of the experiment and the end of each phase. Metagenome and metatranscriptome analysis were conducted at the end of the two final phases.

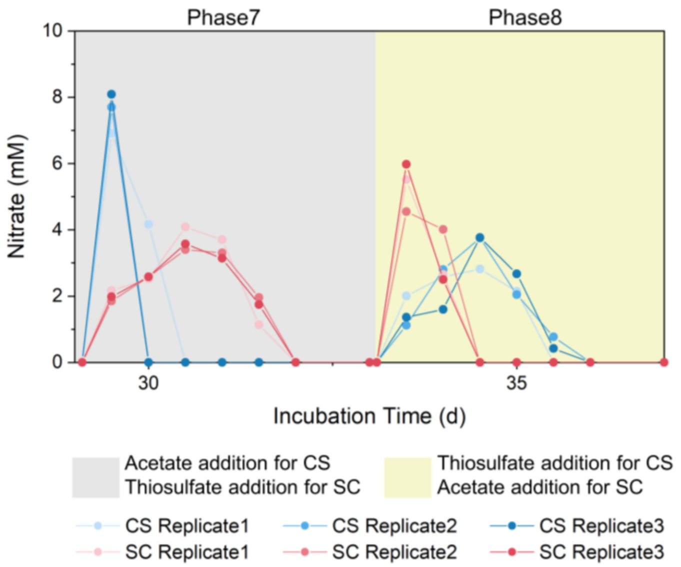

Fig. S2 Nitrate reduction rate in the experiments. Nitrate reduction rate during the last two phases was calculated according to Supplementary Method.

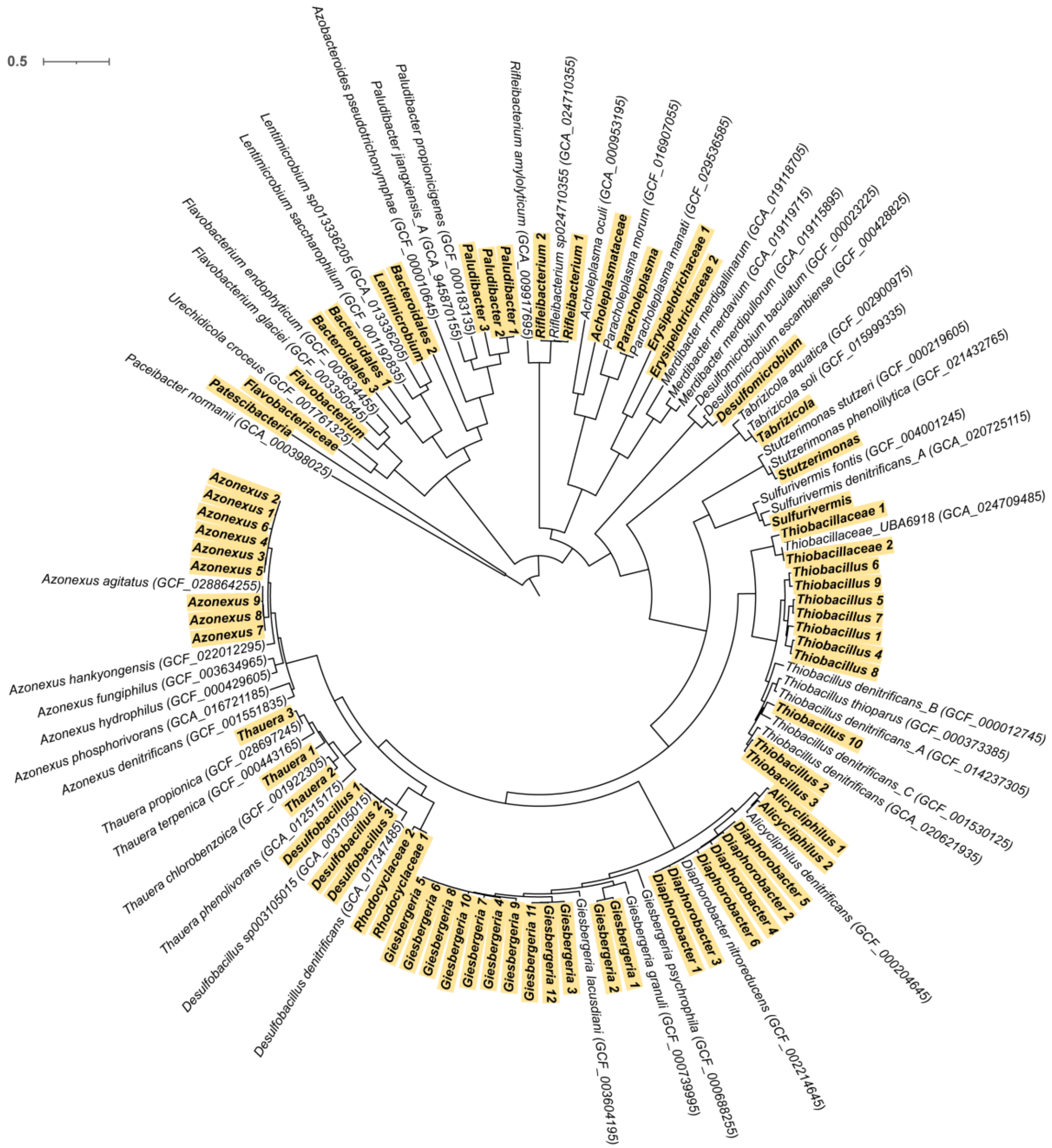

Fig. S3 Phylogeny of enriched populations. Sixty-nine metagenome-assembled-genomes (MAGs) recovered from this study (background in yellow) and forty-eight closely related reference genomes (GTDB IDs in brackets) are included.

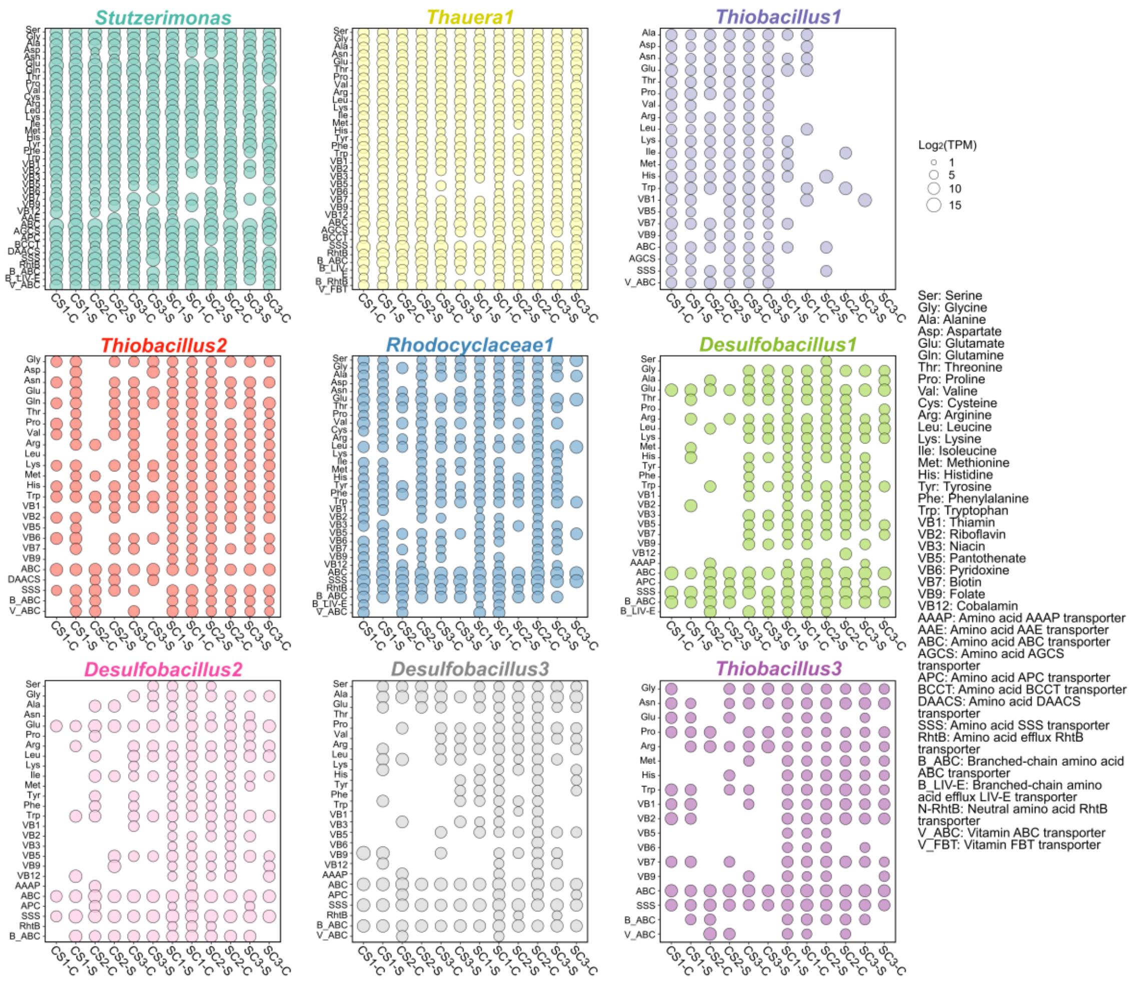

Fig. S4 TPM of genes involved in the biosynthesis and transport of amino acids and vitamins in the ten most abundant populations. A *Patescibacteria* MAG was excluded due to few annotated genes. The genes for biosynthesis are indicated with the names of amino acids or vitamins. The genes involved in transport are indicated with the family name of the transporter.
